# Methicillin-Resistant *Staphylococcus aureus* Infection to a Stent Graft in the Superficial Femoral Artery

**DOI:** 10.1016/j.jaccas.2025.104652

**Published:** 2025-08-20

**Authors:** Mitsukuni Kimura, Nobuhiro Suematsu, Jun Okadome, Hiroyuki Ito, Toru Kubota

**Affiliations:** a Department of Cardiology, Saiseikai Fukuoka General Hospital, Fukuoka, Japan; b Department of Vascular Surgery, Saiseikai Fukuoka General Hospital, Fukuoka, Japan

**Keywords:** endovascular therapy, graft infection, MRSA, peripheral artery disease, Viabahn stent graft

## Abstract

**Background:**

Endovascular therapies for peripheral artery disease have become widespread, but the risk of device infection, though rare, must be considered. Prompt management is essential to preserve the patient's limb and life.

**Case Summary:**

A 79-year-old man presented with intermittent claudication and resting pain in his left leg and underwent multiple endovascular treatments targeting the lesion in the left superficial femoral artery. After treatment, he developed fever, swelling, and pain in his right thigh. Further evaluation revealed an infection of a previously implanted stent graft in the right superficial femoral artery, necessitating surgical removal of the graft.

**Discussion:**

Stent graft infections, though uncommon, can have severe consequences. Prevention and early detection are critical, but diverse patient and lesion backgrounds often complicate this process.

**Take-Home Message:**

This case highlights the potential risks of infections in implanted devices for peripheral artery disease and underscores the importance of proper management.

## Visual Summary

**Figure:**
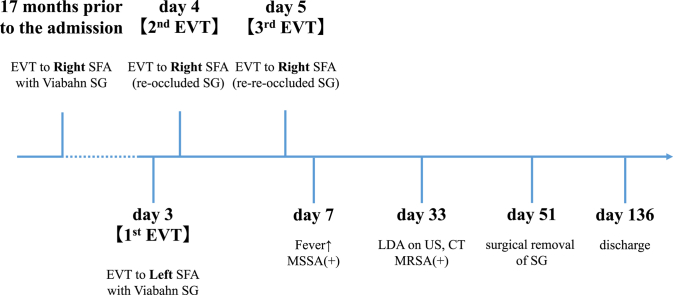

**Visual Summary:**
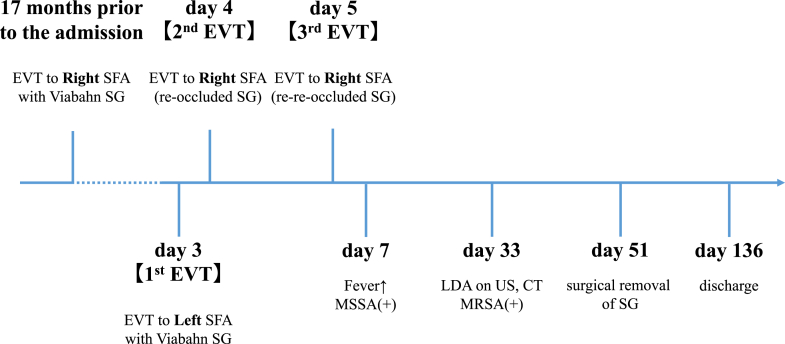
Timeline of Clinical Events and Interventions CT = computed tomography; LDA = low-density area; MRSA = methicillin-resistant *Staphylococcus aureus*; MSSA = methicillin-susceptible *Staphylococcus aureus*; US = ultrasound; other abbreviations as in Figure 1.

## History of Presentation

A 79-year-old man was referred to our institution because of an intermittent claudication and resting pain in his left leg.

**Take-Home Messages**

• In addition to the patient comorbidity such as hemodialysis, diabetes, and wound infection, repeated intervention may increase the risk of infection for implanted devices in patients with peripheral artery disease.
• Surgical excitation is often necessary for the complete healing of peripheral endovascular device infection.

## Medical History

The patient had a history of hypertension, diabetes mellitus on insulin, hemodialysis owing to diabetic nephropathy, and previous endovascular therapy (EVT) in his right superficial femoral artery (SFA) with a Viabahn stent graft (SG) (W. L. Gore & Associates) for intermittent claudication at another hospital 17 months before admission to our institution.

## Investigations

At admission, the patient’s ankle-brachial index was 0.73 (right side)/0.70 (left side), and computed tomography scans showed chronic total occlusion in his left SFA, with patent SG in his right SFA. Despite both exercise and optimal medical treatment, his symptoms had not improved, suggesting the indication for revascularization.

## Management (Medical/Interventions)

**Figure 1:**
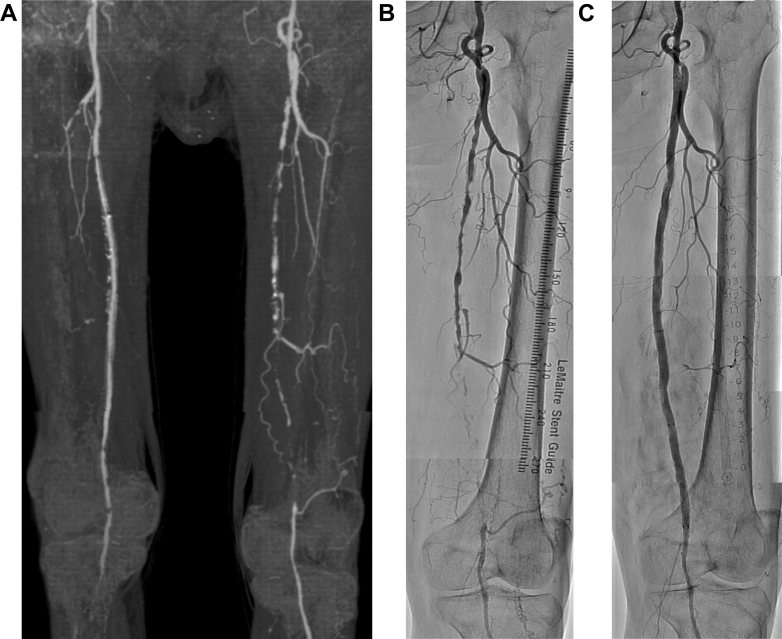
Initial EVT to the Left SFA TASC C lesion with short segment of CTO was treated with 2 SGs, inserted from the right femoral artery. (A) Preprocedural computed tomography findings. (B) Preprocedural and (C) postprocedural angiographic findings of left SFA. CTO = chronic total occlusion; EVT = endovascular therapy; SFA = superficial femoral artery; SG = stent graft; TASC = Transatlantic Inter-Society Consensus.

**Figure 2:**
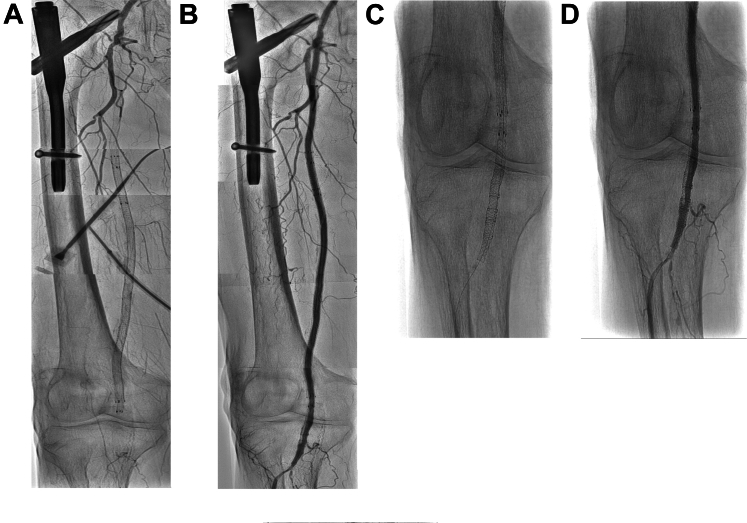
Occluded SGs in the Right SFA Two consecutive EVTs were performed to the right SFA for the reocclusion of the previously implanted SGs at 1 day and 2 days after the initial EVT. (A) Preprocedural and (B) postprocedural angiographic findings of right SFA. (C) Magnified plain image and (D) magnified angiographic image of the distal SFA extending to the arteries below the knee. Abbreviations as in Figure 1.

Initial EVT to the left SFA was performed in a hybrid operating suite. From the right common femoral artery (CFA), locally anaesthetized, a Viabahn SG was percutaneously inserted in the occluded left SFA, as the vessel preparation resulted in severe dissection (Figure 1). Hemostasis at the puncture site was attempted using a Perclose ProGlide Suture-Mediated Closure System (Abbott Vascular) but ultimately failed, necessitating 15 minutes of manual compression. At the conclusion of the procedure, we confirmed that the SG in the right SFA was patent. However, the P3 segment of the popliteal artery was poorly visualized, indicating a possible distal embolization (Video 1). The left-side ankle-brachial index on the following day went up to 0.87, however the right side became unmeasurable, and we subsequently confirmed reocclusion of the implanted SG by ultrasound. Both the previously mentioned possible distal embolization and hemostatic complications may have contributed to the acute reocclusion of the SG. In the same manner as the first EVT, we approached from the left CFA to the right SFA in a crossover fashion (Video 2). The guidewire was able to easily pass the occluded SG, and after thrombectomy and balloon dilation, an additional SG was inserted in the distal part of the implanted SG (Video 3). The patient’s symptoms were relieved after the reintervention, and the total procedure time was 131 minutes. Once again on the following day, we found reocclusion of the SG that had been reopened the previous day. As surgical revascularization was not suitable for the poor quality of the saphenous vein, EVT was adopted for the second time to recanalize the SG. Similar to the previous procedure, the left CFA was percutaneously punctured. The initial angiogram revealed complete occlusion of the SG, with no visible runoff vessels beyond its distal edge (Video 4). Owing to the nationwide discontinuation of urokinase, effective thrombolytic therapy could not be administered. Despite repeated balloon angioplasty and thrombectomy, the below-knee arterial lesion reoccluded multiple times, ultimately necessitating the use of a Supera stent (Abbott Vascular)—an intervention we had initially intended to avoid—which consequently prolonged the procedure time (250 minutes in total) (Figure 2, Video 5). Ultimately, the patient received 3 EVTs in the 3 consecutive days after admission, and all of these procedures were performed without any prophylactic treatment with antibiotics.

**Figure 3:**
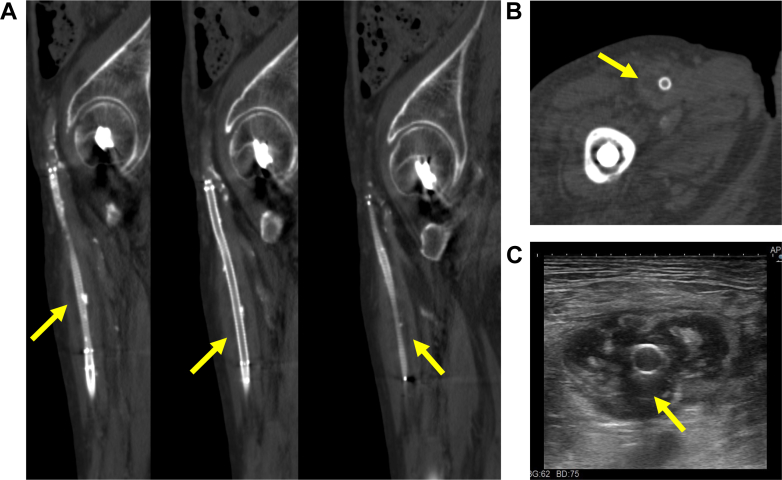
Ultrasound and Computed Tomography Findings CT scans and ultrasound reveal a low-density area around the stent graft (SG) (yellow arrows), suggesting possible abscess formation. (A) Sagittal and (B) cross-sectional CT scans demonstrate a low-density area around the SG. (C) A cross-sectional ultrasound shows an irregular low-echogenic structure surrounding the SG.

**Figure 4:**
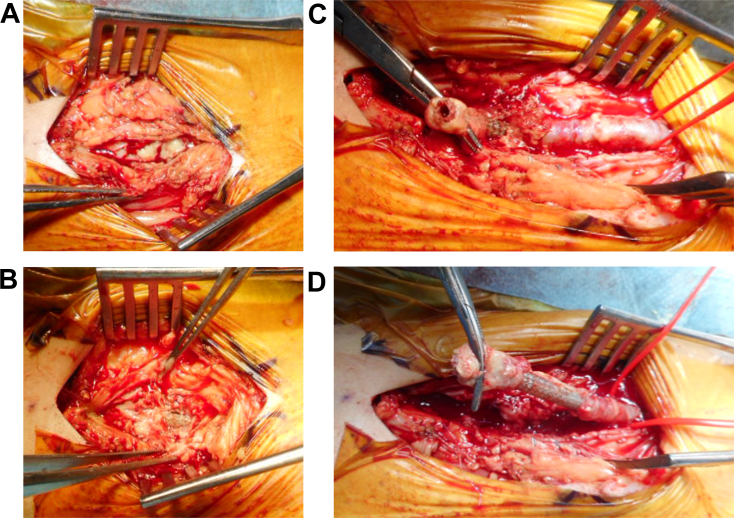
Operative Findings (A) Under local anesthesia with mild sedation, the thigh muscles were exposed, and large amount of purulent drainage and pus was observed behind them. (B) The right SFA was almost completely destroyed by the infection, with the SG visible and no surrounding arterial tissue. (C and D) Both the proximal and the distal parts of the SG were cut and the entire mass was extracted without revascularization of the SFA. Abbreviations as in Figure 1.

**Figure 5:**
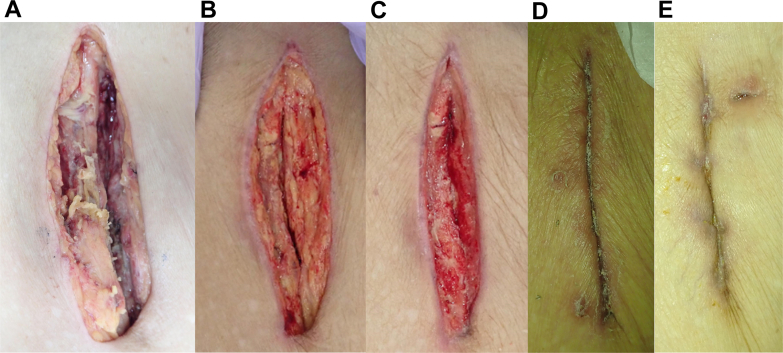
Serial Appearance of the Wound Appearance at (A) 1 day, (B) 3 weeks, (C) 4 weeks, (D) 6 weeks, and (E) 29 weeks after surgical resection.

The day after the 3 consecutive EVTs, the patient had a fever of 38.3 °C with pain and swelling in the left femoral punctual site of the previous EVTs, and blood culture showed methicillin-susceptible *Staphylococcus aureus*. Temporary reduction of fever was achieved during a 2-week period during which ceftriaxone was administered, and blood culture turned out to be negative. However, 3 days after termination of ceftriaxone, the patient noted both pain and swelling in the right thigh again. Ultrasound and computed tomography showed a low-density area around the SG in his right SFA, suggesting possible abscess formation (Figure 3). Methicillin-resistant *S. aureus* (MRSA) was subsequently isolated from the abscess via ultrasound-guided aspiration, and we adopted the surgical resection of the infected SG instead of continuous conservative treatment. Under local anesthesia with mild sedation, the thigh muscles were exposed, and large amount of purulent drainage and pus were observed behind them. The right SFA itself was almost completely destroyed by the infection, with the SG visible and no surrounding arterial tissue (Figure 4). Both the proximal and the distal part of the SG were cut, and the entire mass was extracted without revascularization of the SFA. The patient was discharged 29 weeks after resection of the SG, with complete epithelialization of the wound at the thigh; he presented no resting pain of gangrene on his right leg (Figure 5). There was no evident sign of infective endocarditis or septic embolization to the distal foot during the entire clinical course.

## Outcome and Follow-Up

The patient did not develop any ischemic symptoms in either limb until he died from septic shock due to pneumonia 14 months after extraction of the SG.

## Discussion

With the recent development of therapeutic devices, EVT for complex SFA lesions has been increasingly adopted. According to the latest guidelines of the European Society of Cardiology,^1^ EVT should be the first choice even for complex lesions, especially in surgically high-risk patients. Along with this trend, there also have been case reports regarding various device infections after EVT, including bare-metal stents,^2^ drug-eluting stents,^3^ and covered stents.^4^ As there are a limited number of case reports that describe the background of patients who undergo EVT, it is not clear whether these infections are attributable to the type of device used. To prevent these device-related infectious complications, strategies such as initially finalizing with a drug-coated balloon—thereby avoiding scaffold placement—and employing prophylactic antibiotic therapy after device implantation—which we had not been applying—may be beneficial. As far as we know, there have been few case reports regarding the infection of SGs deployed in the SFA. Schneider et al^4^ reported a similar case, describing the surgical exploration of Viabahn 6.5 months after its deployment in the SFA due to a MRSA infection in a 67-year-old diabetic patient with ischemic ulcer in her foot and heel.

After reviewing 30 previously reported cases, Hogg et al^2^ proposed several risk factors for bare-metal stent infections after EVT, including repeat puncture of the same arterial access site, increased procedure time, passing devices through a previously deployed stent, and multiple interventions on adjacent sites, all of which were also observed in our present case.

The Viabahn SG is a heparin-bonded expanded polytetrafluoroethylene–covered stent having shown a better long-term patency, especially in long SFA lesions.^5^ Before the availability of atherectomy devices, our institution used SGs for the treatment of complex SFA lesions such as chronic total occlusions or heavily calcified segments, particularly in patients undergoing hemodialysis. Compared to bare-metal stents, the microporous polytetrafluoroethylene lining of the Viabahn SG may be vulnerable against stent infection, but there have been no comparative data owing to the limited number of cases regarding device infection. Our case presents an SG infection 17 months after implantation, proposing the hypothesis that the SG had been chronically infected and the additional procedures (ie, the second and the third EVTs) activated the colonization of MRSA.

Among patients with end-stage renal disease, infectious disease, attributed to various physiological abnormalities such as increased oxidative stress, chronic inflammation, endothelial dysfunction, elevated uremic toxins, and disorders of mineral metabolism, is recognized as an important complication.^6^ In a longitudinal cohort study analyzing data from the U.S. Renal Data System, Jaar et al^7^ reported that peripheral vascular disease is an independent risk factor for septicemia in patients with diabetic hemodialysis. Furthermore, even in patients with mild to moderate chronic kidney disease, lower kidney function is known to be associated with a linear and graded risk of infection-related hospitalization.^8^ Patients on hemodialysis have poor prognosis related to peripheral artery disease, and target lesions in the peripheral artery tend to be long and heavily calcified, which make them suitable candidates of SGs for EVT. However, careful surveillance during the perioperative period is important regarding the risk of device infection as related to the aspects we have described.

Patients occasionally note thigh pain after stent implantation, especially if the procedure was aggressive in order to obtain an adequate expansion of vessel wall. It is also true that a certain degree of inflammation (elevated white blood cell or C-reactive protein counts and slight fever) is commonly evident after SG implantation, implying a biological reaction to an artificial component of the device. Therefore, it is sometimes difficult to distinguish these assumable phenomena from reactions against undesirable infection, especially soon after the procedure.

## Conclusions

Infection of an implanted SG in the SFA is rare, but we should take into account its risk, especially in patients on hemodialysis. Once it happens, it could be fatal for the prognosis of the patient’s life and limb. Long-term continuation of antibiotics is not always effective, and surgical extraction of the device with adjunctive femoropopliteal reconstruction is mandatory.

## Funding Support and Author Disclosures

The authors have reported that they have no relationships relevant to the contents of this paper to disclose.

## Appendix

Video 1 Completion Angiogram of the Right SFA Stent Graft Extending Into the Arteries Below the Knee at the End of the First EVT

Video 2 Initial Angiogram of Occluded SFA Stent Graft Extending Into the Arteries Below the Knee During the Second EVT

Video 3 Completion Angiogram Demonstrating the Reopened Right SFA Stent Graft Extending Into the Arteries Below the Knee During the Second EVT

Video 4 Initial Angiogram of Reoccluded SFA Stent Graft Extending Into the Arteries Below the Knee During the Third EVT

Video 5 Completion Angiogram Demonstrating the Reopened Right SFA Stent Graft Extending Into the Arteries Below the Knee During the Third EVT
